# Applying network flow optimisation techniques to minimise cost associated with flood disaster

**DOI:** 10.4102/jamba.v15i1.1444

**Published:** 2023-09-15

**Authors:** Simon D. Okonta, John Olaomi

**Affiliations:** 1 Department of Statistics, School of Applied Sciences and Technology, Delta State Polytechnic, Otefe-Oghara, Nigeria; 2 School of Statistics, College of Science, Engineering and Technology, University of South Africa, Johannesburg, South Africa

**Keywords:** cost minimisation, disaster, flooding, stochastic programming, uncertainty, vulnerability

## Abstract

Flooding disasters in most parts of the world has become worrisome to the government and to the humanitarian emergency organisations. In this article, the authors proffer a mathematical solution to minimise the cost of rescue operations, using stochastic programming of a multicommodity and multimodel network flow. In the formulation, the authors considered four supply depots: national centre depot (NCD), three local distribution centres (LDCs) and six points of distribution (PODs). Two vehicle types were helicopters by air and trucks by land. Three basic types of emergency relief materials include food, water and medical items. Three basic scenarios were mild, medium and severe situations with associated probabilities of 0.25, 0.5 and 0.25, respectively. The formulated model was solved using the LINGO software. The results show that the formulated model effectively reduced the cost of distribution during emergency rescue operation, as there was a thin line between demand and met demand. For the scope of this model, a minimised cost of about $1016673.37 is sufficient to carry out successful rescue operations.

**Contribution:**

The estimated amount of $1016673.37 becomes a benchmark for the government, research agencies and other developmental agencies for the purpose of planning. By using the air and road transport modes, and allowing direct and indirect transportation to the PODs, it saved time, resulting in many lives being saved.

## Introduction

Most often emergencies result from serious, unexpected and dangerous situations such as accidents occasioned by man, major terrorist attacks and natural hazards, which require urgent action (World Health Organization [WHO] 1989). Emergencies that are large scales usually result in destruction of properties and numerous loss of lives (Guha-Sapmir et al. 2011). It has been observed that once large-scale emergencies occurred, such as the flood that happened in Nigeria in 2012 or the earthquake that happened in Japan in March 2011, several damages, loss of lives and large amount of rescue resources are required (Federal Emergency Management Agency [FEMA] 2012). The incidence of natural hazards are, in recent times, increasing. It usually results in massive damage of properties such as infrastructure and transportation networks (Udoh & Anietiok 2015). The United Nations International Strategy for Disaster Reductions (UNISDR 2012) defines a disaster as:

[*A*] sudden, calamitous event that causes serious disruption of the functioning of a community or a society causing widespread human, material, economic and\or environmental losses which exceed the ability of the affected community or society to cope using its own level of resources.

A disaster involves an overwhelming situation that local communities cannot handle, thereby leading to their call for help from national and sometimes international community. The World Health Organization (1989) defined it as:

[*A*]ny occurrence that causes damage, destruction, ecological disruption, loss to human life, human suffering, deteriorating of health as well as health services on a scale sufficient to warrant an extraordinary response from outside the affected community or area.

Furthermore, the Centre of Research on Epidemiology on Disasters (CRED) defines disaster as:

[*A*] situation or event which overwhelms local capacity, necessitating a request to a national or international level for external assistance, an unforeseen and often such event that causes great damages, destructions and human suffering. (Guha-Sapmir et al. 2011)

Nzeribe-George et al. (2014) see disaster as ‘an unforeseen and often sudden event that causes great damages, destruction and human suffering, which are often caused by nature or an anthropogenic force’. In addition to the natural calamities mentioned earlier, many dangerous diseases such as the coronavirus disease 2019 (COVID-19), cholera, dysentery and typhoid spread as an epidemic.

**FIGURE 1:**
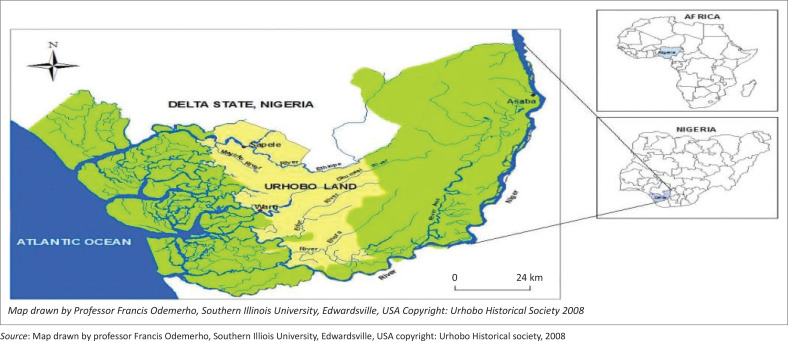
A map of Delta State showing Urhobo land and major rivers of Western Niger Delta.

On the 2023 earthquake in Turkey-Syria, the United Nations Development Programme (UNDP) early estimates are that up to 210 million tonnes of rubble will need to be cleared in Türkiye alone (TurkeyAuthorities 2023). The estimated area of debris is equivalent to an area of 10 km by 10 km – equivalent to 14 000 soccer fields covered in debris piled 1 m high. The destruction has left 1.5 million people homeless and will require the construction of 500 000 new housing units to compensate (TurkeyAuthorities 2023). In Nigeria, flooding is experienced as a major disaster. The reason for this is said to be the rise of sea levels as a result of global warming together with the saturated nature of the wetlands in Nigeria. In the event of flood, the affected citizens are often distorted with their socioeconomic life and livelihood. The effects of flood are devastating and some hardly recover from it. The people in the Delta State are predominantly wildlife habitats and crop farmers. Most times, contaminated flood waters overflow the riverbanks and affect their produce. As alluded to by Mmom and Aifesehi (2013), hunger, famine, disease and epidemic outbreaks are usually resultants of flood. Flood vulnerability is often experienced in low-lying coastal region, deltas and small basins (Japhet 2018) as depicted in Figure 1. All settlements within these regions are vulnerable to flooding; hence, Delta State of Nigeria had suffered flooding for some recent times (Amangabara & Obenade 2015). Greater than 2012 flood disaster is the 2022 disaster. The Director General (DG), National Emergency Management Agency (NEMA) (2022), stated that:

The 2022 flooding is the worst in the history of Nigeria, and that 2022 flood hit the country with devastating consequences, impacted thousands of communities and wreaked havoc in all the 36 States of the Federation and the Federal Capital, Abuja.

A study, which reconstructed the history of floods in KwaZulu-Natal, South Africa, since the 1840s, confirmed a widely held view – yet anecdotal – that the April 2022 floods were likely the most catastrophic natural hazard yet recorded in KwaZulu-Natal and that flooding events have doubled over the last century or more (Stefan & David 2022).

Udoh and Aniefiok (2015) and Okereke (2007) summarised the consequences of flooding to include loss of human lives, submerging of residence and streets, inflow of sewage municipal pollution and health hazards, traffic obstruction, aesthetic discolouring, disruption of services, infrastructural damage and economic loss. In the recent times, many humanitarian agencies and government have shown concern over the flight of those who suffer the impact of flood. Aid logistics and supply chain management have risen to reduce the impact of floods (Japhet 2018). Thomas and Kopezak (2005) defined the process of rescue operations as:

[*T*]he process of planning, implementing, and controlling the efficient, cost-effective flow and storage of goods, materials, as well as related information, from the point of origin to the point of consumption for the purpose of alleviating the suffering of vulnerable people. (pp. 12–13)

The primary desires therefore become utilising the available resources effectively to meet the urgent assignment of saving lives and properties. The authors therefore should agree with Thomas (2003) when he said:

[*L*]ogistics plays a key role in disaster response operations, it serves as a link between procurement and distribution, and between headquarters and the field, and is crucial to the effectiveness and responsiveness to major humanitarian programs such as health, food, shelter, water, and sanitation.

Van Wassenhove (2006) observed that 80% of logistics is required for the efficient and effective relief operations and more precisely the supply chain management, and that such management is immensely important for a successful humanitarian operation. Recognising the immense role of disaster management, various authors agree that it has four distinct phases, which are mitigation, preparedness, response and recovery (Altay & Green 2006; FEMA 2012; Morteza, Abbas & Behnam 2015; Rawls & Turnqkist 2012).

Furthermore, thousands of hectares of farmland were flooded from torrential rains. Dam bursts are a common cause of flooding in Nigeria. Edward-Adebiyi (1997) reported that Ogunpa disaster in Ibadan, Nigeria, which claimed over 200 lives and damaged property worth millions of Naira, was because of urban flooding. Delta State, in particular, Nzeribe-George’s report has it that floods have claimed more lives than any other kind of disaster (Mmom & Aifesehi 2013). They equally added that it has resulted in more destruction of properties. Flooding in Nigeria has driven millions of people from their homes, destroyed businesses and sent academic institutions packing, polluted water resources and increased the risk of diseases (Abam 2006; NEMA 2012). Table 1 showed reports of some flood disasters in Nigeria and the number of people affected.

**TABLE 1:** Floods in Nigeria and the people affected.

Date	Number of people affected
August 1988	300 000
11 September 1994	580 000
10 October 1998	100 000
27 August 2001	84 065
05 September 2003	210 000
10 September 2009	150 000
13 September 2010	1 500 200

Furthermore, the authors also observe the impacts of the economic damages of flood that occurred during 1985–2011 in terms of monetary cost as shown in Table 2.

**TABLE 2:** Floods in Nigeria and the monetary cost.

Date	Cost (US dollar in thousands)
23 September 1985	8000
11 September 1994	66 500
15 August 2000	1900
20 September 2000	4805
27 August 2001	3000
05 September 2003	2570
07 August 2005	147
28 August 2011	30 000
13 September 2011	1500

The need for vendors to cut costs was stressed in a study on robust optimisation using mixed-integer linear programming for the supply chain for liquefied natural gas (LNG) (Arun et al. 2020). The researchers observed that the supply parameters being used were stochastic, hence they classified the parameters as interval-based. To validate their model, they used a CPLEX solver of GAM. They also created a cuckoo optimisation algorithm (COA) to solve their model. The vendor profit and the robust cost are compared and evaluated to find the ideal robustness level. To address the cost issue, Doufour et al. (2018) suggested optimal logistics service network architecture for humanitarian response with the main goal of reducing overall expenses. Using modelling, statistical analysis and optimisation methods, they observed that it was affordable to add a regional distribution hub in Kampala. Their findings indicate that the average cost decrease was around 21%.

**FIGURE 2:**
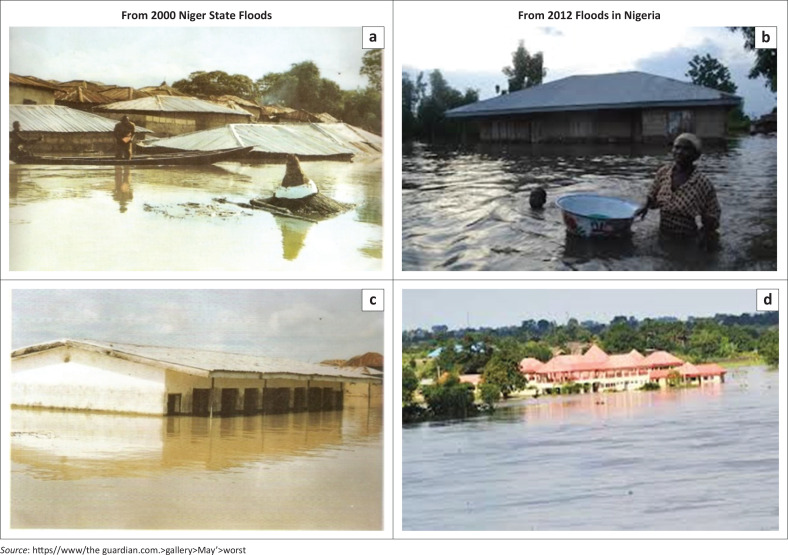
Pictures of flooding disasters in Nigeria.

Now beyond measure, the flood of 2012 in Nigeria is judged to be the worst ever (NEMA 2012). The Nigerian authority is said to contain the initial excess run-off through contingency measures, but in September 2012, the dams are forced open in a bid to relieve the pressure leading to the overflow of the water reservoirs in both Nigeria and neighbouring Cameroon and Niger Republic. The incidence resulted in the destruction of riverbanks, severe loss of property and collapse of social infrastructures, along with the destruction of network of roads, farmlands, crops and livestocks. On September 29, the UN office (UNISDR 2012) reported that the flood had affected 134 371 people, displaced and killed 148. At the end of October, over 7.7 million people had been affected, over 2.1 million displaced, about 363 persons were reported dead and over 618 000 houses were destroyed (Abam 2006). Figure 2 shows the damages.

## Statement of problems

Flood disasters are not new in Nigeria. However, the flood case of 2012 took the nation by surprise and affected 30 of the 36 states in Nigeria. The country was reported to have lost about 500 000 barrels of crude oil output per day because of the severe flooding (Amangabara & Obenade 2015). After a post-disaster need-assessment carried out between November 2012 and March 2013 in conjunction with the World Bank and the Global Facility for Disaster Reduction and Recovery, *The Punch* on 27 May 2013 reported that an unusually heavy rainfall led to severe flooding over nearly the entire country, causing many casualties and massive displacement.

The United Nations, development partners and relevant ministries and agencies put the estimated total value of infrastructure, physical and durable assets destroyed at $9.6billion. The economic activities lost was valued at $7.3bn. The combined value of damages and losses was estimated at $16.9bn (Amangabara & Obenade 2015). This general consequence has been traced to poverty, governmental planning, poor budgeting system, reckless management of fund, lack of insurance, weak institutions, a lack of response preparation and problems with emergency response (*The Punch* 2013).

Despite the challenges and consequences of disasters with their unquantifiable effects, it is obvious that government and other key agencies or institutions that have the role in these vulnerable areas have not risen to the challenge for control. Government makes budget, and some amount of money is allocated to ecological control. It is a common practice to see politicians divert this money without investing it for the purpose. It is also common to see the destruction of forests and mangroves, improper planning of communities resulting in improper settlement of houses and water ways. All these pose a threat to communities’ lives and property. Disaster preparedness for any eventuality in Nigeria and Delta State is still a dream yet to be realised. According to the Pan American Health Organization (PAHO 2013):

[*T*]o ensure an effective preparedness to disaster, there is the need for pro-active planning and collaboration among disaster experts, communicators, or administrators to disaster management, training, teamwork, and investment etc. (PAHO 2013)

On the other hand, Van Wassenhove (2006) and Novia, Hozumi and Tatsuo (2015) said that a strategic approach towards disaster preparation requires a supply-chain wide collaboration.

In recent times, optimisation appears to be a functional technique to solve the rising need of emergency humanitarian logistics in flood-prone areas such as Delta State. Mathematical programming has turned to be an ideal modelling tool to address problems of uncertainty. One such tool is the stochastic programming, which is able to handle the random variables as it concerns flood disasters in Delta State.

## Objective of study

Considering the effects of floods on infrastructure, human lives and well-being, as well as the high costs associated with flood response, there is a need to come up with a model that could be applied to minimise the costs for disaster response. Consequently, the objectives for this study are to: (1) minimise the various cost associated with the entire process of transporting the relief materials from the preposition point to the final consumer at the point of distribution (POD), (2) estimate and guide the government on the yearly ecological budget and (3) prepare an estimate to government on the immediate rehabilitation of the people affected by flooding.

## Model formulation

The model is a stochastic programming that handles uncertainty. Before stating the working objectives, the authors made the following assumptions:

- An inventory may be stored at the national centre depots (NCDs), but when that happened, it is penalised
- A local distribution centre (LDC) may be supplied by either NCD or other LDCs.
- Given that no LDC is open within the area of a POD, such POD may be served by multiple LDCs.
- When disaster occurs, roads or path and/or facility may be damaged or destroyed. This may likely affect the performance ability of suppliers and candidate NCDs.
- At the POD, the cost parameters and the demand levels are stochastic and are likely to be associated with the scenarios of the disasters and the level of impact of the disaster. *N* should be taken as a set of possible disaster situation.
- The relief commodity will be more than one type and each commodity will differ in volume, procurement cost, storage and cost of transportation. The commodities for this model are food, clothes and medical facilities.
- The probability distribution of the scenarios shall be assumed to have been derived by experts in this field of study.
- It is assumed that the transportation cost by air is twice that of by land.
- In some cases, where the supplies and demand parameter of relief commodities differ from the real conditions, estimated information may become useful because of damages, but it will be good estimation for planning.
- The chosen PODs must be away from the disaster zone.

### Sets and/or indices

**Table:** 

I:	Sets of candidate NCDs indexed by i ∈ I
J:	Sets of candidate LDCs indexed by j ∈ J
K:	Sets of demand points in the affected area: POD
L:	Sets of relief material types indexed by l ∈ L
N:	Sets of scenarios indexed by n ∈ N
M:	Sets of vehicles indexed by m ∈ M

### Parameters

**Table:** 

P_n_:	Probability of scenario n.
V_l_:	Volume of relief item 1 per unit.
C^n^_j_:	LDC_j_ capacity under scenario n.
C^n^_j_:	LDC, capacity under scenario n.
Cl_J_:	Capacity of NCD, for item 1.
d^n^_kl_:	Amount of demand at the point k for relief type 1 under scenario n.
Fl_J_:	Fixed cost of running NCD_i_.
F2^n^_j_:	Fixed cost of running LDC_j_.
φ_i_l:	Cost of procuring and holding one unit of item 1 at NCD_i_.
φ^n^_i_l:	Procuring and holding cost for one unit of item 1 at LDC_j_ under scenario n.
s^n^_kl_:	Unit shortage cost of item 1 under scenario n at demand point k.
H_il_:	Maximum amount of supply of item 1 in NCDi, with distribution function ϕ_i_l.
U^n^_il_:	Usable percentage of total amount of item 1 pre-positioned at NCD_i_.
α:	Confidence level, 0 ≤ α ≤ 1.
w:	Service quality proportion.
t_max_:	Maximum allowed delivery duration.
tl^n^_ijk_:	Transportation time from NDC_i_ to demand point k via LDC_j_ under scenario n.
t2^n^_ik_:	Direct transportation time from NCD_i_ to demand point k under scenario n.
a1^n^_ijklm_:	Transportation cost from NCDi to demand point k via LDCj under scenario n.
C2^n^_jklm_:	Cost of transportation one unit of item directly from NCD_i_ to demand point k: POD_k_.
T:	Threshold of coverage.
T_ijk_:	Distance from relief supplier i to k via j.
T_ik_:	Distance from relief supplier i to k directly.
x2^n^_ijkm_:	Type m vehicle assigned from relief supplier i via point j to point k under scenario n (an integer).
x3^n^_ijkm_:	Type m vehicle assigned from relief supplier i directly to affected area under scenario n (an integer).
E1_im_:	Type m vehicle capacity, in relief supplier i.
E2_jm_:	Type m vehicle capacity, in relief supplier j.
E3_m_:	Load capacity vehicle type m.
W_l_:	Average weight of commodity 1.
AP1^n^_ijk_:	A path being available from supplier i to affected area k via point j.
AP2^n^_ik_:	A path being available from supplier i to affected area k directly.

### Decision variables

**Table:** 

B_ij_:	Quantity of item 1 stored at NCD_i_.
X_ijk_:	Quantity of item 1 shipped from NCD_i_ to LDC_j_.
${\text{Y}}_{jkl}^{n}$:	Quantity of item 1 shipped from LDC_j_ to point k under scenario n.
${\text{Z}}_{jkl}^{n}$:	Quantity of item 1 shipped directly from NCD_i_ to point k under scenario n.
${\text{SQ}}_{kl}^{n}$:	Shortage quantity of relief item 1 at point k under scenario.
${\text{X}}_{ijklm}^{n}$:	Quantity of commodity 1 assigned from relief supplier ito affected area k via point j by type m vehicle under scenario n.
${\text{Y}}_{iklm}^{n}$:	Quantity of commodity 1 assigned from relief supplier i to affected area k directly by type m vehicle under scenario n.


$$\text{Y}1_{i}=\{\begin{array}{cc} 1, & \text{if}\;{\text{NCD}}_{i}\;\text{is}\;\text{opened}\\ 0, & \text{otherwise} \end{array}$$ [Eqn 1]

$${\text{M1}}_{j}^{n}=\{\begin{array}{cc} 1, & \text{if}\;{\text{LDC}}_{i}\text{is}\;\text{opened}\;\text{under}\;\text{scenario}\;\text{n,}\\ 0, & \text{otherwise} \end{array}$$ [Eqn 2]

$${\text{B}}_{ijk}^{n}=\{\begin{array}{cc} 1, & \begin{array}{l} \text{if}\;\text{any}\;\text{relief}\;\text{item}\;\text{is}\;\text{shipped}\;\text{from}\;{\text{NCD}}_{i}\;\text{to}\\ \text{demand}\;\text{point}\;\text{k}\;\text{via}\;{\text{LDC}}_{j}\;\text{under}\;\text{scenario}\;\text{n,} \end{array}\\ 0, & \text{otherwise} \end{array}$$ [Eqn 3]

$${\text{ρ}}_{ik}^{n}=\{\begin{array}{cc} 1, & \begin{array}{l} \text{if}\;\text{any}\;\text{relief}\;\text{item}\;\text{is}\;\text{shipped}\;\text{directly}\;\text{from}\;{\text{NCD}}_{i}\;\text{to}\\ \text{demand}\;\text{point}\;\text{k}\;\text{via}\;{\text{LDC}}_{j}\;\text{under}\;\text{scenario}\;\text{n,} \end{array}\\ 0, & \text{otherwise} \end{array}$$ [Eqn 4]

$${\text{γ}}^{n}=\{\begin{array}{cc} 1, & \text{if}\;\text{scenario}\;\text{n}\;\text{is}\;\text{included}\;\text{in}\;\text{a}\;\text{reliability}\;\text{set}\\ 0, & \text{otherwise} \end{array}$$ [Eqn 5]

$$n_{ik}^{n}=\{\begin{array}{cc} 1, & \text{if}\;\text{path}\;\text{is}\;\text{available}\;\text{from}\;\text{relief}\;\text{supplier}\;\text{i}\;\text{to}\;\text{the}\;\text{affected}\;\text{area}\;\text{k}\;\text{directly}\;\text{to}\;{\text{LDC}}_{i}\;\text{under}\;\text{scenario}\;\text{n,}\\ 0, & \text{otherwise} \end{array}$$ [Eqn 6]

Furthermore, let us assume:

${\text{AA}}_{ijk}^{n}$: Available distance from relief supply i to affected area k via point j under the scenario n.

${\text{B}}_{ik}^{n}$: Available distance from relief supply i to affected area k directly under the scenario n.

${\text{Zd}}_{kl}^{n}$: Quantity of unmet demand for commodity 1 in affected area k under the scenario n.

### The model


$$f=Min\sum_{i}F1iyi+P_{n}\left[\sum_{j}\sum_{n}F2_{j}^{n}M1_{j}^{n}+\sum_{k}\sum_{l}\sum_{n}SQ_{kl}^{n}s_{kl}^{n}+\sum_{i}\sum_{j}\sum_{k}\sum_{l}\sum_{m}\sum_{n}a1_{ijklm}^{n}X_{ijklm}^{n}+\sum_{i}\sum_{k}\sum_{l}\sum_{m}\sum_{n}a2_{iklm}^{n}Y_{iklm}^{n}\right]+\sum_{i}\sum_{l}\phi_{il}{\text{β}}_{il}$$ [Eqn 7]

Subject to:


$$d_{kl}^{n}-\sum_{i}\sum_{j}\left(X_{ijklm}^{n}\right)-\sum_{i}\left(Y_{iklm}^{n}\right)=S_{kn}^{n},\forall i,l,m,n$$ [Eqn 8]

$$U_{il}^{n}H_{il}\geq \sum_{j}\sum_{k}\left(X_{ijklm}^{n}\right)-\sum_{k}\left(Y_{iklm}^{n}\right),\;\forall k,l,m,n$$ [Eqn 9]

$$H_{il}\leq C1_{il}y1,\;\forall i,l$$ [Eqn 10]

$$\sum_{i}\sum_{k}\sum_{l}\sum_{m}X_{ijklm}^{n}V_{1}\leq C_{j}^{m}M1_{j}^{n},\forall j,n$$ [Eqn 11]

$$\sum P^{n}{\text{γ}}^{n}\geq \propto$$ [Eqn 12]

$$S_{kl}^{n}\leq d_{kl}^{n}\left(1-{\text{γ}}^{n}\right),\forall k,l,n$$ [Eqn 13]

$$x1_{im}\leq \sum E1_{im}Yl,\forall i,m$$ [Eqn 14]

$$\sum_{k}\sum_{j}x2_{ijkm}^{n}+\sum_{k}x3_{ikm}^{n}\leq x1_{im},\forall i$$ [Eqn 15]

$$\sum_{j}w_{l}X_{ijklm}^{n}\leq E3x2_{kjim}^{n},\forall i,i,j,k,m,n$$ [Eqn 16]

$$\sum_{i}w_{l}Y_{iklm}^{n}\leq E3x3_{kjim}^{n},\forall i,k,m,n$$ [Eqn 17]

$$\sum_{i}\sum_{k}\sum_{m}X_{ijklm}^{n}+\sum_{i}\sum_{m}Y_{iklm}^{n}\leq d_{kl}^{n},\forall i,l,n$$ [Eqn 18]

$$Zd_{kl}^{n}=d_{kl}^{n}-\left(\sum_{i}\sum_{j}\sum_{m}X_{jklm}^{n}+\sum_{l}\sum_{m}Y_{iklm}^{n}\right),\forall k,l,n$$ [Eqn 19]

$$t1_{ijk}^{n}{\text{β}}_{ijk}^{n}\leq t_{\max},\forall i,j,k,n$$ [Eqn 20]

$$t2_{ik}^{n}{\text{ρ}}_{ik}^{n}\leq t_{\max},\forall i,j,k,n$$ [Eqn 21]

$$\sum_{l}\sum_{m}X_{ijklm}^{n}\leq MI{\text{β}}_{ijk}^{n},\forall i,j,k,n$$ [Eqn 22]

$$\sum_{i}\sum_{m}Y_{iklm}^{n}\leq MI{\text{ρ}}_{lk}^{n},\forall i,k,n$$ [Eqn 23]

$$AA_{ijk}^{n}=\{\begin{array}{c} T_{ijk},AP1_{ik}^{n}=1\\ +\infty ,AP1_{ijk}^{n}=0,\forall i,j,k,n \end{array}$$ [Eqn 24]

$$BB_{ik}^{n}=\{\begin{array}{c} T_{ik},AP2_{ik}^{n}=1\\ +\infty ,AP2_{ik}^{n}=0,\forall i,k,n \end{array}$$ [Eqn 25]

$$x2_{ijkm}^{n}=\{\begin{array}{cc} \geq 0 & AA_{ijk}^{n}\leq T\\ =0 & AP1_{ijk}^{n}>T,\forall i,j,k,m,n \end{array}$$ [Eqn 26]

$$x3_{ikm}^{n}=\{\begin{array}{cc} \geq 0 & BB_{ik}^{n}\leq T\\ =0 & BB_{ik}^{n}>T,\forall i,k,m,n \end{array}$$ [Eqn 27]

y1,  ∈ (0,1,)∀i [Eqn 28]

$${\text{x1}}_{\text{im}}\geq 0,\;\text{an}\;\text{integer,}\;\forall \text{i,}\;\text{m}$$ [Eqn 29]

$${\text{x2}}_{\text{ijkm}}^{\text{n}}\geq 0,\;\text{an}\;\text{integer,}\;\forall \text{i,}\;\text{j,}\;\text{k,}\;\text{m,}\;\text{n}$$ [Eqn 30]

$${\text{x3}}_{\text{ikm}}^{\text{n}}\geq 0,\;\text{an}\;\text{integer,}\;\forall \text{i,}\;\text{k,}\;\text{m,}\;\text{n}$$ [Eqn 31]

### Description of the constraints

Here, the authors are considering an objective optimisation model design to solve emergency allocation network problem with:

- Multisupplier
- Multirelief items
- Multivehicle
- Multiaffected areas

The objective, which is equation (7), is about the minimisation of the total cost involved in the relief allocation process. It explains the level of economy involved.

The constraint equation (8) explains that the shortfall of item 1 at the demand point k is the difference between the amount of item 1 demanded at point k and the amount of item 1 transported both directly and indirectly to point k. Constraint (Eqn 9) shows at scenario n, the total amount of relief material 1, which is shipped directly and indirectly from NCD_i_, cannot exceed the total usable amount of relief material 1, which is stored in NCD_i_. Constraint (Eqn 10) is to ensure that the items of relief material 1, which is stored in NCD_i_ do not exceed its capacity. It further ensures that shipment from NCD_i_ can only happen if NCD_i_ is opened. Constraint (Eqn 11) explains that not all the LDCs_i_ need to be open before it can receive relief materials from NCD_i_. Furthermore, any relief material coming from NCD_i_ to LDC_j_ must not exceed its capacity. It cannot store relief material above its capacity. Constraint (Eqn 12) establishes that the allocated relief material does not exceed the amount supply. This constraint is defined as a chance constraint to be able to handle the uncertainty inherent in the supply of relief materials within a defined confidence level, close to 1. Constraint (Eqn 13) assures that if a shortage is associated with, it is zero. Constraint (Eqn 14) defines the capacity limits of vehicles in the relief supplier centre. Vehicles should only gather at the NCDi where the relief supplier is available. The constraint (Eqn 15) demands that the number of vehicles at work should not exceed the supplier’s actual capacity. Therefore, the number of vehicles both for direct and indirect shipments cannot exceed the capacity of the supplier. Constraints (Eqn 16) and (Eqn 17) check the load capacity limits of the vehicles and enhance the free flow of the commodity at both indirect and direct shipments, which should not exceed the amount of demand. Constraint (Eqn 18) tells us the relationship between the allocation amount and demand. It shows that allocation must not exceed the amount of demand. Constraint (Eqn 19) defines the unmet demand. Constraints (Eqn 20) and (Eqn 21) are concerns with maximum delivery time. Constraints (Eqn 22) and (Eqn 23) are complementary to (Eqn 20) and (Eqn 21), respectively. The relationship between (Eqn 20) and (Eqn 22) is the same as (Eqn 21) and (Eqn 23). Constraint (Eqn 21) is an indirect route with shipment from NCD_i_ to POD_k_ via LDC_j_ under different scenarios using binary variables. Constraint (Eqn 23) is the direct shipment. Constraint (Eqn 24) guarantees the availability of path. When the path is destroyed, the distance available will be infinite for indirect shipment. The same applies to constraint (Eqn 25) in the case of direct shipment. Constraints (Eqn 26) and (Eqn 27) define the limits of coverage, while constraints (Eqn 28–30) define the exact domains for the decision variables.

## Research methods and design

**FIGURE 3:**
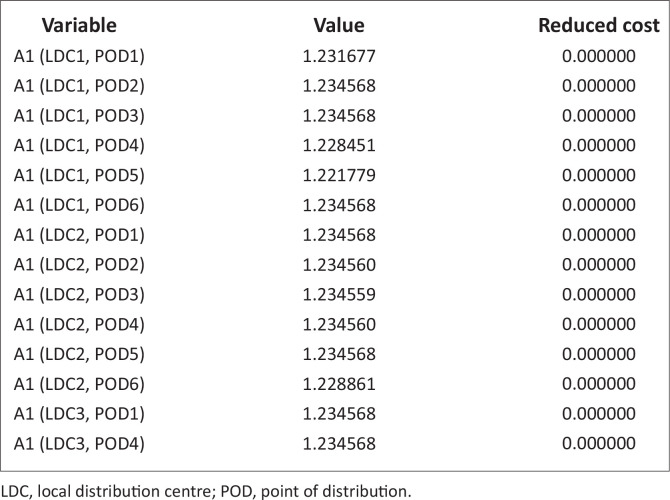
Transportation cost (A1).

**FIGURE 4:**
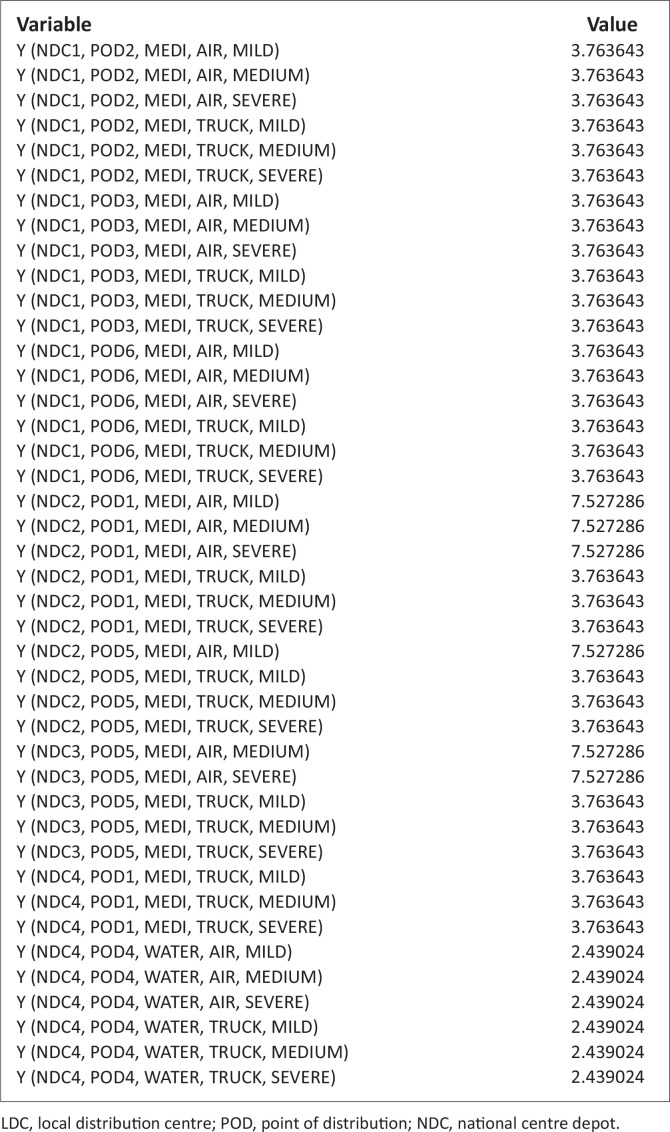
Quantity of items assigned directly from national centre depots to points of distributions by vehicle transportation $\left({\text{Y}}_{\text{iklm}}^{\text{n}}\right)$.

These problems, because of the randomness inherent in it, are nonlinear. It is noteworthy that there are commercial software designs to solve such nonlinear problems. The authors have therefore employed LINGO software (Lindo Software 2020), which has its peculiarity in language, symbols and syntax. The necessary data and/or information collected were inserted into this model (Eqn 7), with the constraints specified in equations [8–21], using the software; the results are stated in Table 4 and Table 5 and Figure 3 and Figure 4.

**TABLE 3:** Making use of towns/communities as our national centre depots (NCD), local distribution centres (LDC) and points of distribution (PODS).

NCD	LDC	PODS
Asaba	Urhobo	Sapele
Warri		Abraka
Ughelli	Ukwuani	Kwale
Agbor		Aboh
	Isoko	Emevo
		Uzere

LDC, local distribution centre; POD, point of distribution; NDC, national centre depot.

**TABLE 4:** Probability of the scenarios (0.25, 0.50 and 0.25) on the cost of distribution to the points of distributions.

Distribution from LDCs to PODs	Cost × 10^3^
LDC1, POD1	1.234567
LDC1, POD2	1.234568
LDC1, POD3	1.234568
LDC1, POD4	1.234567
LDC1, POD5	1.234567
LDC1, POD6	1.234568
LDC2, POD1	1.234568
LDC2, POD2	1.234232
LDC2, POD3	1.234018
LDC2, POD4	1.234565
LDC2, POD5	1.234568
LDC2, POD6	1.234443
LDC3, POD1	1.234568
LDC3, POD2	1.233388
LDC3, POD3	1.233806
LDC3, POD4	1.234568
LDC3, POD5	1.234566
LDC3, POD6	1.234566

LDC, local distribution centres; POD, point of distribution.

**TABLE 5:** Probability of the scenarios (0.25, 0.25 and 0.50) on the cost of distribution to the points of distributions.

Distribution from LDCs to PODs	Cost × 10^3^
LDC1, POD1	1.234560
LDC1, POD2	1.234568
LDC1, POD3	1.234568
LDC1, POD4	1.234561
LDC1, POD5	1.234560
LDC1, POD6	1.234568
LDC2, POD1	1.234568
LDC2, POD2	1.234564
LDC2, POD3	1.234564
LDC2, POD4	1.234563
LDC2, POD5	1.234568
LDC2, POD6	1.234564
LDC3, POD1	1.234568
LDC3, POD2	0.000000
LDC3, POD3	0.000000
LDC3, POD4	1.234568
LDC3, POD5	0.000000
LDC3, POD6	0.000000

LDC, local distribution centre; POD, point of distribution.

### Case description

This work used Delta State as the area of study. The State has the Niger Basin with many rivers and tributaries. Many people live near the riverbank.

The authors consider four supplies depots: NCD, three LDCs and six PODs. The model comprised vehicle types such as:

*Air (helicopters)
*Land (trucks)

The maximum amount of supply of item l is treated in NCD_i_.

Three types of emergency supply items shall be food, clothes and medical facilities. Three scenarios (mild, medium and severe) should be considered with associated probabilities of 0.25, 0.5 and 0.25, respectively. These probabilities are assumed to be derived by experts. The transport cost is linear cost function of distance assuming the air transport to be twice expensive as the land transport. Therefore, the data about distance from the emergency facilities are provided in tables within the present prevailing circumstances with reflection on the rescue operation during the 2012 flood disaster in Nigeria. Some, however, may be estimated as near reality by experts.

Let n = n_1_, n_2_ and n_3_ represent mild, medium and severe scenarios, respectively. Let the weight function of the scenario n be P(n), satisfying 0 ≤ P(n) ≤ 1. It should however be observed that this probability usually depends on: (1) the type of disaster, (2) the intensity of the disaster and (3) the environmental factors. The travel trip is a function of the impact of the disaster in the region. For the land trucks, their travel time within the region is determined by the nature of the routes. Bozorgi-Amiri and Khorsi (2015) said, ‘The set of act-able routes is determined according to experts, each starting at a supplier and traversing a sequence of RDCs’. This case study considered the following towns and/or communities (Table 3) as NCDs, LDCs and PODs.

## Results

It is a recognisable truth that where the issue of life and death is the crucial matter forming the agenda of the mind, the issue of cost becomes less crucial. However, it is important to consider budget limits. This research work considered the various costs associated with the rescue operations at an emergency situation: the cost of direct operations and that of the indirect rescue operations. The various costs considered include the fixed cost at the NCDs and the LDCs; the indirect transport cost and direct transport cost; the shortage cost and the holding cost. The total cost derived was $1016673.37. This figure becomes very necessary for the government, research agencies and other developmental agencies for the purpose of planning.

### Quantity of items assigned from national centre depots to points of distributions via local distribution centres by vehicle transportation

Figure 5(a,b and c) and Figure 6. This figure explains the distribution of relief materials from the NCDs to the PODs via the various LDCs using a particular mode of transportation. Any particular NCD can serve any particular POD depending on the availability of road network with a suitable mode of transportation at a particular scenario. This available option has facilitated the distribution of relief materials given the various options available at each point in time. It is seen that each of the relief materials could be available at the PODs in a reasonable quantity to meet the average needs of the affected community. Figure 6 shows that there are several alternatives in the supply of the relief materials, hence the clumsy nature of the figure.

**FIGURE 5a:**
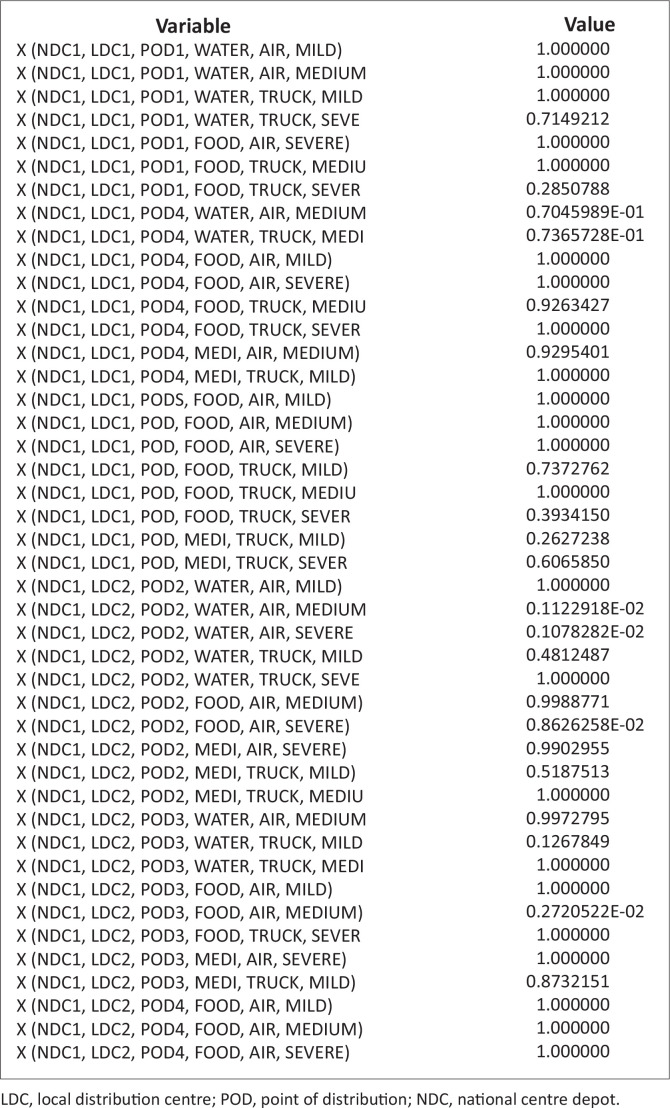
Quantity of Items assigned from national centre depots to points of distributions via local distribution centres by vehicle transportation [(Xijklm^n)].

**FIGURE 5b:**
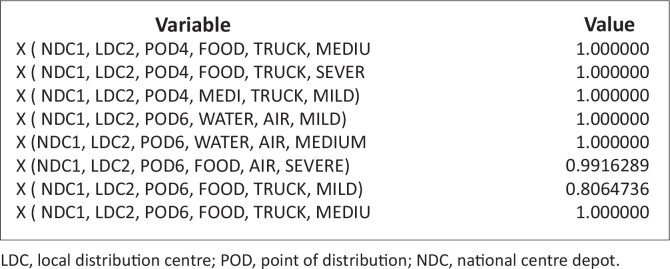
Quantity of items assigned from national centre depots to points of distributions via local distribution centres by vehicle transportation [(Xijklm^n)].

**FIGURE 5c:**
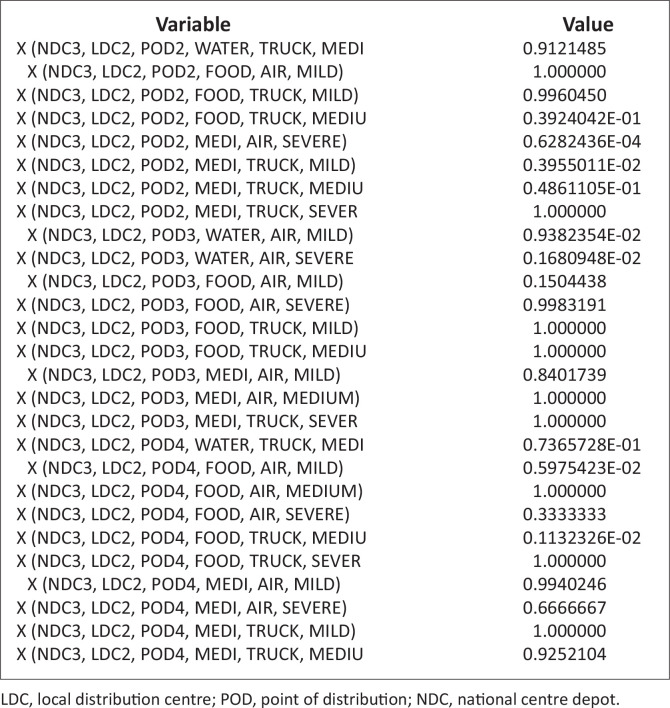
Quantity of items assigned from national centre depots to points of distributions via local distribution centres by vehicle transportation [(Xijklm^n)].

**FIGURE 6:**
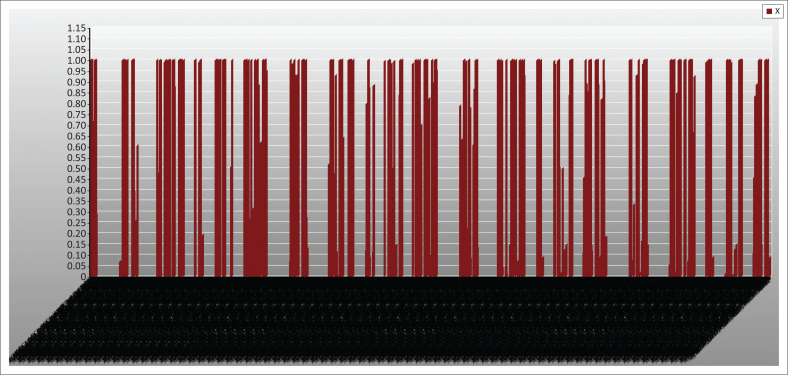
A display of quantity of items assigned from national centre depots to points of distributions via local distribution centres (indirect).

### Quantity of items assigned from national centre depots to points of distributions by vehicle transportation

**FIGURE 7:**
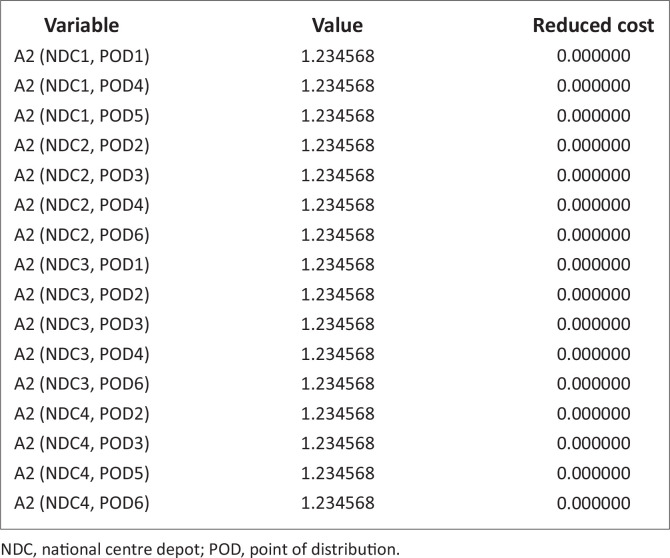
Transportation cost (A2).

Figures 3 and Figure 7 depicts the associated indirect and direct cost respectively, while Figures 6 and Figure 4 depicts the distribution of relief materials from the NCDs directly to the PODs by the possible mode of transportation at a particular scenario. Using this method facilitates distribution of relief materials in a reasonable manner meeting basic needs. It is seen that at a particular scenario, air transport transverses a particular NCD to a POD, and at another scenario it is truck that could be used. This method could help greatly in equitable distribution of relief materials. The summary cost are listed in Box 1.

BOX 1 Summary cost.

**Table:** 

Variable	Price
Fixed cost for NDCS (F1)	$630000.00
Fixed cost for LDCS (F2)	$330820.00
Transportation cost (A1)	$17256.42
Transportation cost (A2)	$17283.95
Holding cost (PHI1)	$14842.00
Shortage cost (S)	$6471.00
**Total**	**$1016673.37**

**Probabilities of the scenario**

Here, the effect of the scenario probabilities was separately analysed because this is the random effect considered in the stochastic model. Kelle, Helmut and Huizhi (2014) while analysing the expected cost minimisation and worst-case scenario in emergency supply noticed that:

[*F*]or the P-reliable criteria solution, as P increases, extreme scenarios with small probabilities are dominating the allocation of resources increasing the cost of transportation for scenarios with higher probability and thus increasing the expected total cost of transportation

Changing individual scenario probabilities (and normalising the others to add up to 1) has more effect on the small probability scenarios.

However, this research has an interwoven effect as the probabilities vary. It must be observed that the authors did not subject their analysis on P-reliable criteria solution. It might be hard to capture all the changes for the different cases; however, the authors will attempt to summarise the effect on cost.

**FIGURE 8:**
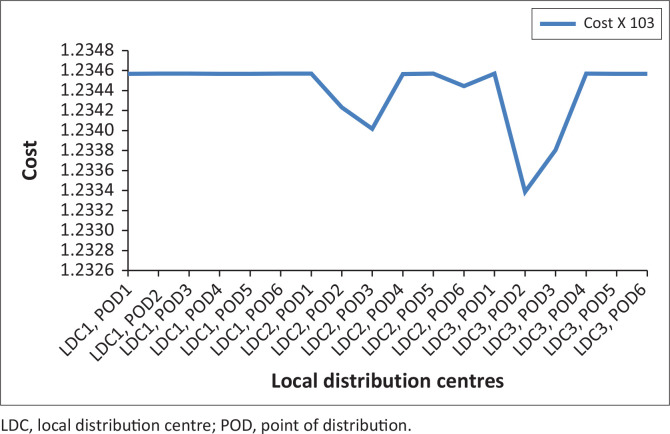
Probability at 0.25, 0.50 and 0.25.

From Table 4 and Figure 8, there is higher probability on the middle that is on the medium scenario. Here, a higher cost effect is experienced on the mild and severe scenarios. Most times, transport logistics affect the cost experience in each of the scenarios. At mild scenario, availability of pre-position materials is a major hindrance. While at severe scenario, poor network of access roads and inadequate communication and information is a hindrance.

Considering this probability variations, with higher probability as the severe scenario, it is observed that cost is higher on the scenario with lower probabilities. The mild and the medium scenarios experiences higher cost of transportation and distribution of relief materials.

This table and figure depicts higher probability at the mild scenario and lower probability at the medium and severe scenarios. This case showed some zero cost as the LDC_1_ and LDC_3_, a case of ‘reduce cost’ situation. This has been discussed in detail in the next section.

The focus of this study is on the effect of variation of probabilities on direct expected cost. The authors wish to introduce the concept of reduce cost. Reduce cost value for each decision variable tells us how much the objective function value will change for a one-unit increase in the decision variable. The reduce cost column gives, for each variable which is currently zero, an estimate of how much the objective function will change if variable is to be non-zero. It is the column referred to as the opportunity cost for the variable.

**FIGURE 9:**
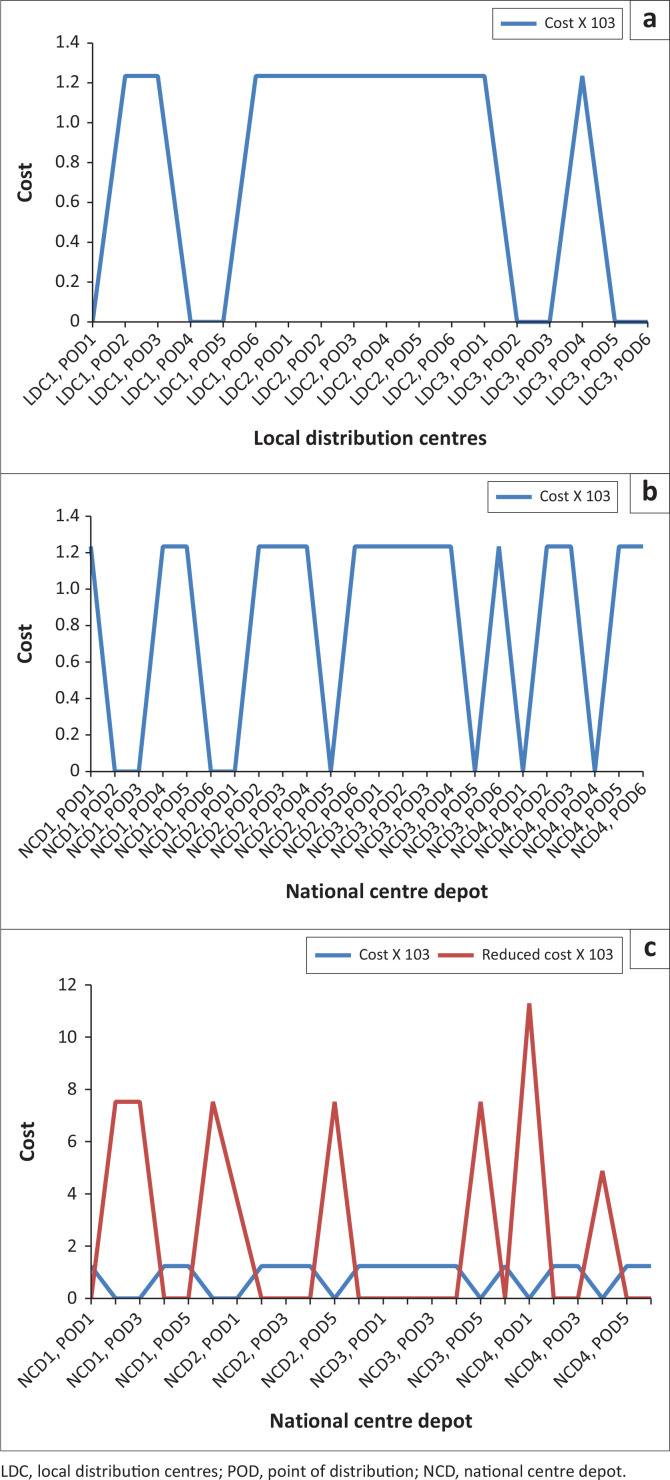
(a) Probability at 0.50, 0.25 and 0.25. (b) Probability at 0.50, 0.25 and 0.25 (direct). (c) With reduced cost.

Table 6 and Figure 9 (a) are indirect distribution while Table 7, 8 and Figure 9 (b) are direct distributions, the authors noticed that at the mild scenario with higher probability, investment on relief materials should be increased by at least 7527.286 units to minimise the effect of transportation cost as it affects direct movement from NCD_1_ to the PODs. At (NCD_4_, POD_1_) and (NCD_4_, POD_4_), the opportunity cost of investing on improved relief materials by 112909.3 units and 4878.049 units, respectively, is minimisation of the direct cost effect of transportation.

**TABLE 6:** Probability of the scenarios (0.50, 0.25 and 0.25) on the cost of distribution to the points of distribution (indirect distribution).

Distribution from LDCs to PODs	Cost × 10^3^
LDC1, POD1	0.000000
LDC1, POD2	1.234568
LDC1, POD3	1.234568
LDC1, POD4	0.000000
LDC1, POD5	0.000000
LDC1, POD6	1.234568
LDC2, POD1	1.234568
LDC2, POD2	1.234567
LDC2, POD3	1.234566
LDC2, POD4	1.234566
LDC2, POD5	1.234568
LDC2, POD6	1.234566
LDC3, POD1	1.234568
LDC3, POD2	0.000000
LDC3, POD3	0.000000
LDC3, POD4	1.234568
LDC3, POD5	0.000000
LDC3, POD6	0.000000

LDC, local distribution centre; POD, point of distribution.

**TABLE 7:** Probability of the scenarios (0.50, 0.25 and 0.25) on the cost of distribution to the points of distributions (direct distribution).

Distribution from NCDs to PODs	Cost × 10^3^	Reduced cost × 10^3^
NCD1, POD1	1.234568	0.000000
NCD1, POD2	0.000000	7.527286
NCD1, POD3	0.000000	7.527286
NCD1, POD4	1.234568	0.000000
NCD1, POD5	1.234568	0.000000
NCD1, POD6	0.000000	7.527286
NCD2, POD1	0.000000	3.763643
NCD2, POD2	1.234568	0.000000
NCD2, POD3	1.234568	0.000000
NCD2, POD4	1.234568	0.000000
NCD2, POD5	0.000000	7.527286
NCD2, POD6	1.234568	0.000000
NCD3, POD1	1.234568	0.000000
NCD3, POD2	1.234568	0.000000
NCD3, POD3	1.234568	0.000000
NCD3, POD4	1.234568	0.000000
NCD3, POD5	0.000000	7.527286
NCD3, POD6	1.234568	0.000000
NCD4, POD1	0.000000	11.29093
NCD4, POD2	1.234568	0.000000
NCD4, POD3	1.234568	0.000000
NCD4, POD4	0.000000	4.878049
NCD4, POD5	1.234568	0.000000
NCD4, POD6	1.234568	0.000000

NCD, national centre depot; POD, point of distribution.

**TABLE 8:** Shortage quantity of item shipped.

Items	Demand	Met demand	Shortage
POD1WATER	6970	6966	4
POD1FOOD	8600	8595	5
POD1MED	4000	4000	0
POD2WATER	5650	5646	4
POD2FOOD	9130	9122	8
POD2MED	3200	3196	4
POD3WATER	4500	4496	4
POD3FOOD	5600	5596	4
POD3MED	1200	1192	8
POD4WATER	2340	2338	2
POD4FOOD	3240	3232	8
POD4MED	890	884	6
POD5WATER	3450	3448	2
POD5FOOD	5760	5752	8
POD5MED	2110	2104	6
POD6WATER	4560	4557	3
POD6FOOD	6765	6753	12
POD6MED	2150	2148	2

POD, point of distribution.

**FIGURE 10:**
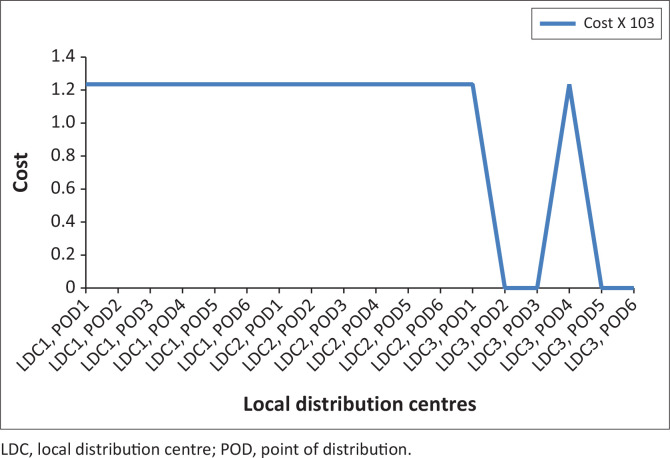
Probability at 0.25, 0.25 and 0.50.

**FIGURE 11:**
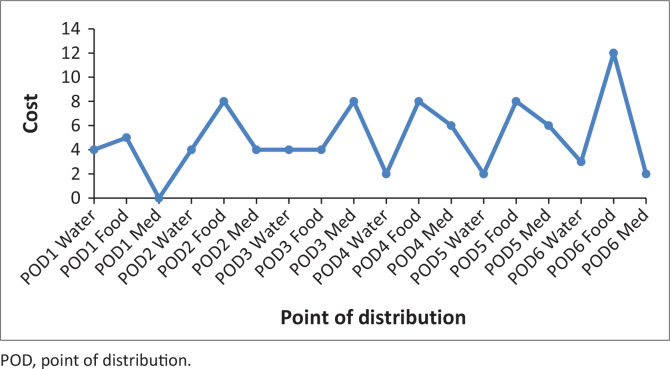
Shortage cost.

Figure 10 considered higher probabilities at the severe scenario and Figure 11 depicts the shortage. As mentioned previously, various costs considered include fixed cost at the NCDs and LDCs, the indirect and direct transport cost, the shortage cost and the holding cost. Generally, the higher the pre-position of materials, particularly at the NCDs, the better it is for the decision makers. This on its own has an increasing effect on holding cost and also the danger of wastage for the perishable items. Often, these contribute to the shortage at the POD.

## Conclusion

From this work, it is evident that the impact of flood in Nigeria and in Delta State, in particular, transcends significantly in an alarming proportion. Flood affects wildlife habitats and crops production and reduces farm produce. The preparation for emergency rescue operations is generally inadequate.

Floods are on a threatening proportion, resulting to loss of many lives and properties. Millions of dollars were spent to deal with flood response. Furthermore, it has been realised that the millions of dollars allocated yearly during budget presentation for ecological fund are not adequately accounted. There is thus an urgent need to effectively plan well to avert these flood threats facing the entire country.

The model has proved to be efficient and effective as a solution to the flood situation in Nigeria. In emergency humanitarian logistics problems, the knowledge of various cost involved helps in making budget. This article considered the costs and the shortage costs and was able to present a minimum cost using mathematical models, which considered uncertainty situation.

## Recommendation

Based on this study, the authors however wish to recommend that:

- Humanitarian relief organisations should adopt more innovative ways of achieving internal control, to reduce cost wastage and massive corruption.
- Humanitarian logistics management should adopt a collaboration venture with international financial administration for proper execution of emergency situation.
- Shortages could be reduced to a minimal level if adequate funding is channelled to provision of warehouses stocked with relief materials that are not perishable for quick response emergencies.
- Government should compensate the people whose wildlife habitat and crops were destroyed.
- This model, using the air transport mode and road transport mode, together allowing direct and indirect transportation to the PODs saved time, resulting in many lives being saved. It enhances minimisation of cost. Further work can be carried out on minimisation of time in the humanitarian logistics planning.

## Data Availability

Data availability can be obtained from the authors on request.
